# Neurotrophins and Trk Neurotrophin Receptors in the Retina of Adult Killifish (*Nothobranchius guentheri*)

**DOI:** 10.3390/ijms25052732

**Published:** 2024-02-27

**Authors:** Caterina Porcino, Kamel Mhalhel, Marilena Briglia, Marzio Cometa, Maria Cristina Guerrera, Patrizia Germana Germanà, Giuseppe Montalbano, Maria Levanti, Rosaria Laurà, Francesco Abbate, Antonino Germanà, Marialuisa Aragona

**Affiliations:** Zebrafish Neuromorphology Lab, Department of Veterinary Sciences, University of Messina, 98168 Messina, Italy; caterina.porcino@unime.it (C.P.); kamel.mhalhel@unime.it (K.M.); marilena.briglia@unime.it (M.B.); marzio.cometa@unime.it (M.C.); mariacristina.guerrera@unime.it (M.C.G.); pgermana@unime.it (P.G.G.); gmontalbano@unime.it (G.M.); mblevanti@unime.it (M.L.); laurar@unime.it (R.L.); abbatef@unime.it (F.A.); mlaragona@unime.it (M.A.)

**Keywords:** neurotrophins, Trks, retina, *N. guentheri*, translational medicine

## Abstract

Specific subpopulations of neurons in nerve and sensory systems must be developed and maintained, and this is accomplished in significant part by neurotrophins (NTs) and the signaling receptors on which they act, called tyrosine protein kinase receptors (Trks). The neurotrophins–tyrosine protein kinase receptors (NTs/Trks) system is involved in sensory organ regulation, including the visual system. An NTs/Trks system alteration is associated with neurodegeneration related to aging and diseases, including retinal pathologies. An emergent model in the field of translational medicine, for instance, in aging study, is the annual killifish belonging to the Nothobranchius genus, thanks to its short lifespan. Members of this genus, such as *Nothobranchius guentheri*, and humans share a similar retinal stratigraphy. Nevertheless, according to the authors’ knowledge, the occurrence and distribution of the NTs/Trks system in the retina of *N. guentheri* has never been investigated before. Therefore, the present study aimed to localize neurotrophin BDNF, NGF, and NT-3 and TrkA, TrkB, and TrkC receptors in the *N. guentheri* retina using the immunofluorescence method. The present investigation demonstrates, for the first time, the occurrence of the NTs/Trks system in *N. guentheri* retina and, consequently, the potential key role of these proteins in the biology and survival of the retinal cells.

## 1. Introduction

The visual system of vertebrates supports interaction with the environment and is crucial for vital processes such as reproduction, migration, food search, and physical activity [1]. In both mammals and fish, visual system performance relies on specialized cells [2] under the control of neurotrophins (NTs) and their specific receptors. Neurotrophins are growth factors involved in the development, maintenance, and neuronal plasticity of different neuronal subpopulations of the central and peripheral nervous system [3,4,5,6,7].

The limitation of neurotrophin amounts during development controls the number of surviving neurons to ensure a match between neurons and the need for the adequate innervation density of the target. Neurotrophins also regulate decisions about cell fate, axon growth, dendrite growth and pruning, and protein expression, such as ion channels, biosynthetic transmitter enzymes, and neuropeptide transmitters which are essential for normal neuronal function [8]. The availability of neurotrophins is also required in adulthood, where it controls synaptic function and plasticity and supports neuronal cell survival, morphology, and differentiation [9].

Moreover, their role has been observed in the regenerative events of sensory epithelia of teleosts, including zebrafish [10,11]. There are two signal transduction systems that support the biological functions of neurotrophins determined by interactions with two types of receptors: the high affinity Trk receptors (tyrosine kinase receptors) and the low affinity neurotrophin P75 receptor (p75NTR). Three main types are known as transmembrane tyrosine kinase proteins, TrkA (tyrosine protein kinase receptors type A), TrkB (tyrosine protein kinase receptors type B), and TrkC (tyrosine protein kinase receptors type C) [5,12]. The recognition and interaction with the substrate occur in a specific but not exclusive way. TrkA is a receptor for the nerve growth factor (NGF), TrkB binds both brain-derived neurotrophic factor (BDNF) and neurotrophin-4 (NT-4), and TrkC recognizes neurotrophin-3 (NT-3). TrkA and TrkB can also interact, with a lower affinity, with NT-3. Furthermore, the p75 receptor can bind to unprocessed or mature neurotrophin and act as a Trks coreceptor [13]. By interacting with their receptors, neurotrophins play a regulatory role in neuronal proliferation, development, survival, growth, differentiation, and synaptic plasticity [4,5,6]. As a highly differentiated neuroectodermal tissue, retina maintenance is governed by neurotrophin-receptor systems. NGF, BDNF, and NT-3 play distinct and crucial roles in the generation of retinal neurons. For instance, NGF and its receptor are expressed in rat retinal ganglion cells where they play a role during development [14,15].

Specifically, it determines the provisional quantity of newly generated neurons in the retina inducing cell death in the developing retina by activating the neurotrophin receptor p75 [16,17].

Neurotrophins, particularly BDNF, play a crucial role in the structural and functional development of retinal ganglion cells, guiding morphological differentiation and controlling the functional adaptability of visual circuits [18].

In vitro studies have shown that BDNF significantly enhances neurite regeneration in the human retina [19]. Furthermore, the TrkB/BDNF signaling pathway regulates the commitment to and/or differentiation of photoreceptor cells from retinal progenitor cells, guiding and controlling cell fate decisions [20]. NT-3 supports neuron differentiation and sustains the survival of differentiated retinal ganglion and amacrine cells during a distinct post-differentiation period [21].

Microscopic examinations showed that the retina consists of distinct layers of nerve cell bodies and two layers of synapses in all vertebrates, from fish to mammals. Although less complex, fish share many anatomical and physiological characteristics with mammals, including humans [22,23,24,25].

Hence, they are an important additional element to consider in mammalian model research [26]. As a matter of fact, the teleost retina shares a lot of anatomical similarities with the mammalian one. For instance, the retina of the teleost *Nothobranchius guentheri* shows a clear-layered structure with distinct cells layers: retinal pigment epithelium (RPE), inner segment/outer segment of the photoreceptor layer (PRL), outer nuclear layer (ONL), outer plexiform layer (OPL), inner nuclear layer (INL), inner plexiform layer (IPL), and ganglion cell layer (GCL). The ONL shows differentiated photoreceptors with well-developed outer segments suggesting the high quality of the vision of this species of fish [1].

*N. guentheri* belongs to the Nothobranchiidae family, a large group of fish, typical of North Africa where they mainly inhabit shallow ephemeral pools and seasonal swamps. The adaptation to this kind of habitat influenced visual system development. For instance, the embryogenesis study of *Nothobranchius* revealed that the duration of its visual system development can be influenced by diapause that is fundamental in ephemeral ponds. However, the crucial steps of this process are similar in other teleosts [27]. Moreover, the adaptation to the natural environment has meant that the visual system of *N. guentheri* is already functional at the hatching moment [28]. Nevertheless, the differentiation of retinal cells does not stop after hatching but continues throughout life [29,30]. The development of new cells in the fish retina occurs from the proliferation of multipotent progenitor cells located in the ciliary marginal zone (CMZ) [31,32] and from the division of Müller glial cells [32]. Finally, the ecological adaptability of *Nothobranchius* to life in shallow waters influenced the thickness of retinal layers [33]. Indeed, the thickness of the temporal, nasal, and cranial areas of the retina are possibly associated with peripheral vision, which plays a fundamental role in various behavioral acts. Some differences between young and old *Nothobranchius* occur. For instance, aged fish show a thinning of the retina layers and a decrease in the pigment epithelium layer [1]. More specifically, some authors [1,34] demonstrated that in older fish, GCLIPL and INL become thinner because of the significant decrease in the number of GCL and INL cells [1]. The species of the genus *Nothobranchius* have a short life expectancy, both in the wild and in captivity [35], and they hold the record for the fastest maturing vertebrate with a short life circle [35]. Indeed, maximum life expectancies range from 3 to 28 months depending on the species. *Nothobranchius* embryo storage is inexpensive, commercially available, and easily reared in captivity. Moreover, the isolation of vertebrate aging-related genes is easy by homology cloning, so it is suitable for test manipulation on aging [36,37,38,39]. The short lifespan allows to perform long-life experiments that are unthinkable in other vertebrates, and the fish of this genus offers the possibility to track the process of tissue aging thanks their different age-related biomarkers [40,41]. In addition, the *Nothobranchius* retina undergoes neurogenesis and regeneration phenomena, even in postnatal life [24], thanks to the persistence of stem cells. For all these reasons, the *Nothobranchius* is being established as a model organism in aging research [42], which has been impaired for a long time in vertebrates due to the lack of short-lived models [36]. *N. guentheri* is not only a suitable object of study in the fields of evolution and development, but has recently also been proposed as a model for studying the structure, formation, and stages of the onset of age-related changes in the visual system [1].

Aging is a complex phenomenon that depends on the interaction of numerous genes, cell pathways, and environmental risk factors. Among the feared complications of aging, there is the age-related degeneration of the retina. Retinal neurons are sensitive to age-related neurodegenerative events. Indeed, it has been demonstrated that alterations and the loss of neuronal cells occur in the aging retina of different experimental models (zebrafish, mice, rats) and humans [43,44,45,46,47,48,49,50,51,52,53,54,55]. The retina is an integral part of the CNS, so it is an intriguing model for studying neurodegenerative phenomena [56,57,58]. However, the aging mechanisms of the killifish retinal system are still poorly understood, and it’s unclear whether changes to the neurotrophin-receptor system could play a role in this process. To shed some light on this aspect, it is necessary to start from the investigation of the occurrence of neurotrophins and their specific receptors in the retina of a model organism for aging studies.

Hence, the present study aims to localize Neurotrophins and tyrosine neurotrophin receptors in the different retinal layers of adult *N. guentheri*.

## 2. Results

In order to analyze the localization of neurotrophin/tyrosine kinase receptors (NTs/Trks) system (brain-derived neurotrophic factor/tyrosine protein kinase receptors type B (BDNF/TrkB), nerve growth factor/tyrosine protein kinase receptors type A (NGF/TrkA), and neurotrophin-3/tyrosine protein kinase receptors type C (NT-3/TrkC), an immunohistochemistry study was conducted. The cells that are immunoreactive to the neurotrophin/receptors system have been identified using a topographic approach and using anti-Opsin, anti-Chat, Parvalbumin, and s100p antibodies as specific markers. The observed immunoreaction showed no differences between samples of different sexes. In this perspective, male retinal images will be shown in the present work.

### 2.1. Histology of N. guentheri Retina

**Figure 1:**
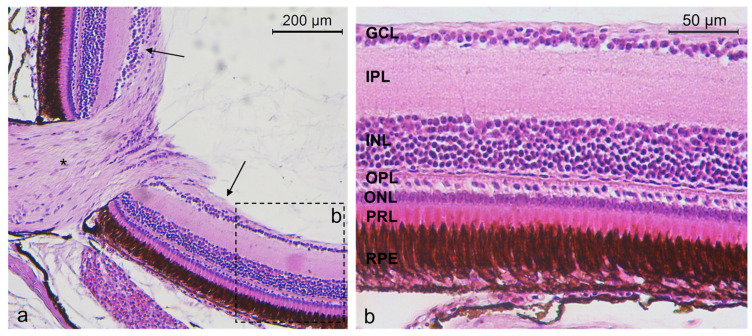
(**a**) *N. guentheri* retina (arrows), optic nerve (asterisk). (**b**) Stratigraphy of *N. guentheri* retina: RPE, PRL, ONL, OPL, INL, IPL, GCL. Hematoxylin-Eosin. Magnification (**a**) 20× (**b**) 40×.

According to the morphological investigation, the retina of *Nothobranchius guentheri* had a similar stratigraphy to other vertebrates. The retina of *N. guentheri* was made up of seven layers: retinal pigment epithelium (RPE), photoreceptor layer (PRL) containing inner and outer segment of rods and cones, outer nuclear layer (ONL) containing cells body of rods and cones, inner nuclear layer (INL) containing different subpopulation of amacrine cells (ACs), bipolar cells (BCs), and horizontal cells (HCs), the GCL containing ganglion cells, the outer plexiform layer (OPL) containing the cellular prolongation between ONL and INL, and the inner plexiform layer (IPL) with the cellular prolongation between INL and ganglion cell layer (GCL) (Figure 1).

### 2.2. Trks Immunofluorescences in N. guentheri Retina

**Figure 2:**
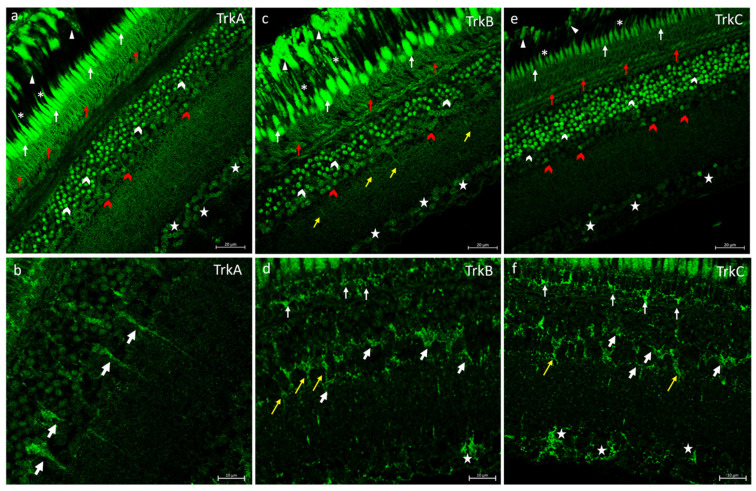
Trks immunostaining in *N. guentheri* retina. (**a**) TrkA-immunoreactivity in the cytoplasmic prolongations of the cells of the RPE (arrowheads); in the inner segment (arrows) and outer segment (asterisk) of the photoreceptors layer; in the OPL (red arrows); in different subpopulation of ACs (white and red gallon-arrows); in the soma of GCs (stars). (**b**) BCs TrkA immunopositive (white bold arrows). (**c**) TrkB immunoreactivity in the cytoplasmic prolongations of the cells of the RPE (arrowheads); in the inner segment (arrows) and outer segment (asterisks) of the photoreceptors layer; in the OPL (red arrows); in several subpopulation of ACs (white and red gallon-arrows); in cellular prolongation in IPL (yellow arrows); in the soma of GCs (stars). (**d**) TrkB immunostaining in the inner segment of PRL (arrows indicate the soma of rods and cones); in BCs (yellow arrows); in the HCs (white bold cells), and in GCs (stars). (**e**) TrkC immunoreactivity in the cytoplasmic prolongations of the cells of the RPE (arrowheads); in the inner segment (arrows) and outer segment (asterisk) of the photoreceptors layer; in the OPL (red arrows); in the INL several subpopulations of ACs (white and red gallon-arrows) and, in the soma of GCs (stars). (**f**) TrkC immunostaining in the inner segment of PRL (arrows indicate the soma of rods and cones); in the BCs (yellow arrows); in the HCs (white bold cells), and in the GCs (stars). (**a**–**c**) Magnification 40×; Scale bar 20 µm. (**d**–**f**) Magnification 63×; scale bar 10 µm.

In the retina of *N. guentheri*, the neurotrophins receptors TrkA, TrkB, and TrkC were always localized in the extensions of RPE and in the inner and outer segments of the PRL (Figure 2a–d,f). In the OPL TrkA, TrkB, and TrkC were found (Figure 2a–c). In the INL, several subpopulations of ACs and some BCs were immunopositive to Trks (tyrosine protein kinase receptors) (Figure 2). Also, the neurotrophin receptor TrkB and TrkC were localized in HCs (Figure 2d,f). Different subpopulations of GCs immunostained to Trk receptors (Figure 2) were also observed.

### 2.3. Double Immunofluorescences of BDNF and TrkB in N. guentheri Retina

**Figure 3:**
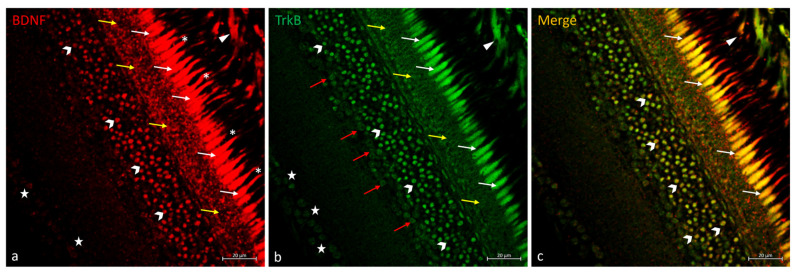
BDNF/TrkB immunostaining in *N. guentheri* retina. (**a**) BDNF-immunoreactivity in the cytoplasmic prolongations of the cells of the RPE (arrowheads); in the inner segment (white-arrows) and outer segment of PRL (asterisk); in the OPL (yellow-arrows); in a subpopulation of ACs (gallon-arrows); weakly stained in the soma of GCs (stars). (**b**) TrkB-immunoreactivity in the cytoplasmic prolongations of the cells of the RPE (arrowheads); in the inner segment of PRL (white-arrows); in the OPL (yellow-arrows); in different subpopulations of ACs (gallon-arrows and red-arrows); in the soma of GCs (stars) poorly stained. (**c**) Colocalization view of BDNF and TrkB in the cytoplasmic prolongations of the cells of the RPE (arrowheads); in the inner segment of PRL (white-arrows); in a subpopulation of ACs (gallon-arrows). Some ACs and GCs TrkB immunopositive did not show double staining with BDNF. A similar condition was observed for the OPL: BDNF and TrkB immunostained but non-overlapping. Magnification 40×. Scale bar 20 µm.

In the retina of *N. guentheri*, the BDNF/TrkB system was immunolocalized in RPE prolongations, and in the inner segment of the PRL (Figure 3). In the PRL, the outer segment was exclusively BDNF immunostained (Figure 3a), and the inner segment of the photoreceptors is BDNF and TrkB was double-marked (Figure 3c). BDNF and TrkB were observed in the OPL, but they did not overlap (Figure 3). Distinct subpopulations of ACs exhibited immunoreactivity to BDNF and TrkB separately (Figure 3a,b), just some of them showed double staining (Figure 3c). In the GCL, the soma of the GCs (ganglion cells) was BDNF and TrkB immunostained (Figure 3a,b).

### 2.4. Double Immunofluorescences of NGF and TrkA in N. guentheri Retina

**Figure 4:**
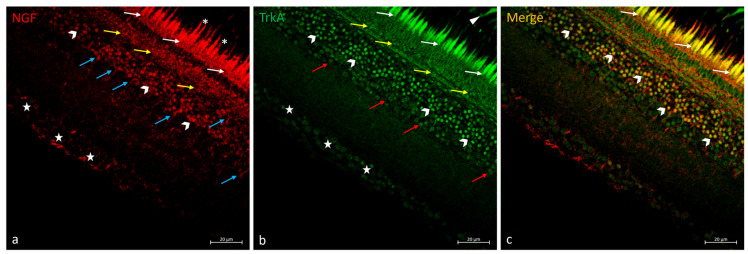
NGF/TrkA immunostaining in *N. guentheri* retina. (**a**) NGF-immunoreactivity in the inner segment (white-arrows) and outer segment (asterisk) of the photoreceptors layer; in the OPL (yellow arrows); in a subpopulation of ACs (gallon-arrows); in the BCs (blue arrows); in the soma of GCs (stars). (**b**) TrkA-immunoreactivity in the cytoplasmic prolongations of the RPE (arrowheads); in the inner segment of PRL (white-arrows); in the OPL (yellow-arrows); in different subpopulation of ACs (gallon-arrows and red-arrows); in the soma of GCs (stars) poorly stained. (**c**) Colocalization view of NGF and TrkA in the inner segment of PRL (white-arrows) and in a subpopulation of ACs (gallon-arrows). Some ACs and GCs TrkA immunopositive did not show double staining with NGF. A similar condition was observed for the OPL: NGF and TrkA immunostained but non-overlapping. Magnification 40×. Scale bar 20 µm.

Research on the NGF/TrkA system in the retina *N. guentheri* showed RPE extensions are immunopositive to the neurotropin receptor TrkA but not to NGF (Figure 4). In the photoreceptor layer, the outer segment labeled NGF and the double stained inner segment NGF e TrkA were observed (Figure 4). NGF and TrkA were immunolocalized in the OPL (Figure 4b), but merging has not been observed (Figure 4c). In the INL, several subpopulations of ACs were immunopositive to NGF and TrkA (Figure 4a,b), some of these immunopositive exclusively to TrkA (Figure 4b) and some of these colocalized (Figure 4c). BCs immunoreactive to NGF were found (Figure 4a). Moreover, different subpopulations of GCs were NGF and TrkA immunostained (Figure 4a,b) in GCL, and no colocalization views were observed (Figure 4c).

### 2.5. Double Immunofluorescences of NT-3 and TrkC in N. guentheri Retina

**Figure 5:**
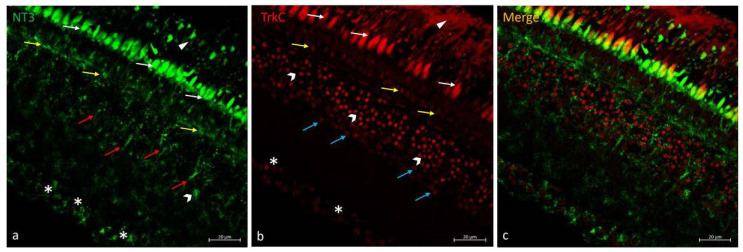
NT-3/TrkC immunostaining in *N. guentheri* retina. (**a**) NT-3-immunoreactivity in the cytoplasmic prolongations of the cells of the RPE (arrowheads); in the inner segment of PRL (white arrows); in OPL (yellow-arrows); in the BCs (red arrows); in the soma of GCs (asterisks). (**b**) TrkC-immunoreactivity in the cytoplasmic prolongations of the cells of the RPE (arrowheads); in the outer segment of PRL (white-arrows), in the OPL (yellow-arrows); in different subpopulations of ACs (gallon-arrows and blue-arrows); in the soma of GCs (asterisks) poorly stained. (**c**) No colocalization view was observed. Magnification 40×. Scale bar 20 µm.

Regarding the NT-3/TrkC system in the *N. guentheri* retina, RPE cell prolongation immunostain was found. In the PRL, the inner segment immunopositive to NT-3 and the outer segment immunoreactive to TrkC have been observed. The OPL was NT-3 and TrkC immunoreactive (Figure 5). In the INL, BCs immunoreactive with NT-3 (Figure 5a) and a subpopulation of ACs immunopositive to TrkC (Figure 5b) were seen. In GCL, several subpopulations of GCs were NT-3 and TrkC immunostained, respectively (Figure 5a,b). Colocalization views were never observed (Figure 5c).

### 2.6. Immunofluorescences of Anti-Opsin, Anti-Chat, Parvalbumin and s100p in N. guentheri Retina

**Figure 6:**
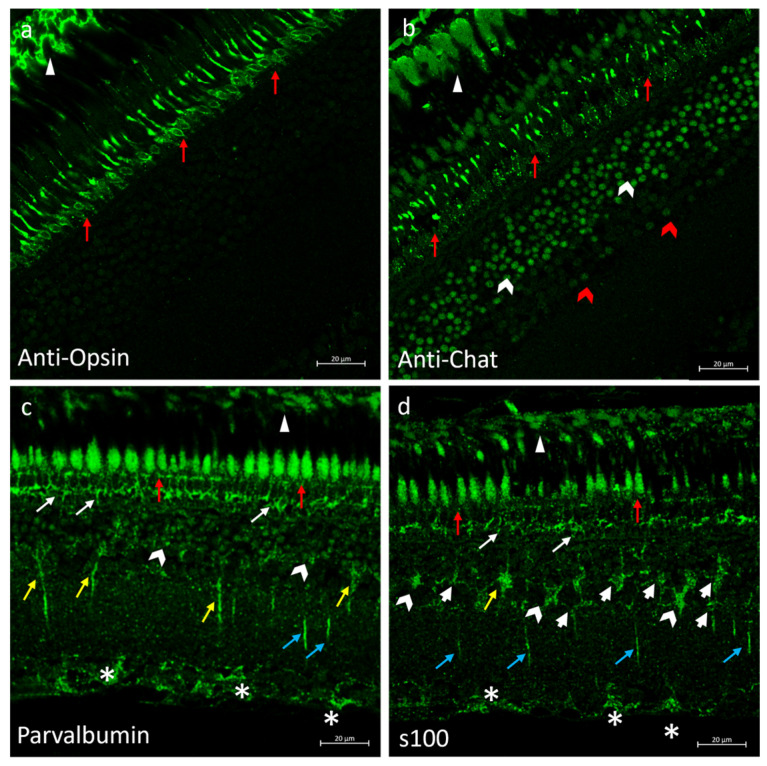
Anti-opsin, anti-chat, parvalbumin, and s100 as specific markers of subpopulation cells in *N. guentheri* retina. (**a**) Anti-Opsin immunoreactivity in the cytoplasmatic prolongation of RPE (arrowheads) and in the rods (red arrows) of PRL. (**b**) Anti-chat immunoreactivity in the cytoplasmatic prolongation of RPE (arrowheads); in the outer segment of the photoreceptor layer (red arrows); in different subpopulations of ACs (white and red gallon-arrows). (**c**) Parvalbumin immunoreactivity in the cytoplasmatic prolongation of RPE (arrowheads); in the outer segment (red arrows) and inner segment (white arrows) of PRL; in the ACs (gallon-arrows); in the BCs (yellow arrows) of INL; in the cellular prolongation in IPL (blue arrows); in the soma of GCs (asterisk). (**d**) s100p immunoreactivity in the cytoplasmatic prolongation of RPE (arrowheads); in the outer segment (red arrows) and inner segment (white arrows) in PRL; in the BCs (yellow arrows), HCs (white bold arrows), ACs (gallon arrows) in the INL; in cellular prolongation of the IPL (blue arrows); in the soma of GCs (asterisk). Magnification 40×. Scale bar 20 µm.

To ascertain the cellular identity of the immunopositive cells shown, anti-Opsin (specific for rods), anti-Chat (specific for ACs), parvalbumin (specific for BCs), and s100p (specific for HCs and GCs) antibody immunoreactions were investigated. The observed immunostaining perfectly overlapped the specificity of the antibodies used as markers (Figure 6). In addition, the RPE was immunoreactive to anti-chat, anti-opsin, s100, and parvalbumin; the outer segment of the PRL was anti-chat, parvalbumin, and s100 immunostained, and the inner segment of the PRL was parvalbumin and s100 immunoreactive. In the INL, ACs were parvalbumin and s100 immunolabeled; finally, GCs were also parvalbumin immunopositive (Figure 6).

### 2.7. Quantitative Analysis

**Figure 7:**
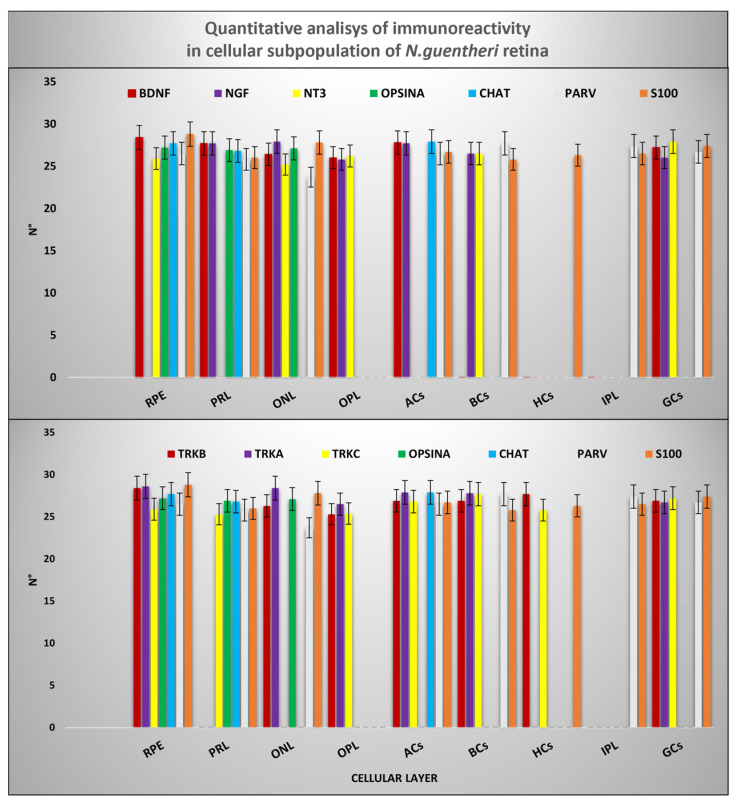
Graphical representation of immunoreactivity quantitative analysis in: RPE, PRL (inner and outer segments), OPL, ACs, BCs, HCs, IPL, and GCs detected by BDNF, NGF, NT-3, TrkA, TrkB, TrkC, anti-Opsin, anti-Chat, Parvalbumin and, s100p. The statistical analysis shows a different distribution pattern of the antibodies used in this study in cellular layer of *N. guentheri* retina. N°: mean of retinal layer cells immunopositive to BDNF, NGF, NT-3, TrkA, TrkB, TrkC, anti-Opsin, anti-Chat, Parvalbumin and, s100p.

According to the results of the quantitative analysis, the cellular prolongations of the RPE were immunoreactive to neurotrophin BDNF and NT-3 and to neurotrophin receptors type A, B, and C. The inner segment of the PRL was always immunopositive to BDNF/TrkB, NGF/TrkA, and the NT-3/TrkC systems; however, the outer segment of the PRL was BDNF and NGF immunoreactive. In the INL, several subpopulations of ACs immunopositive to neurotrophin BDNF and NGF and to Trks (type A, B, and C) were observed. BCs were immunoreactive to Trks (A, B, and C) and to neurotrophins NGF and NT-3, whereas HCs were exclusively immunostained by TrkB and TrkC. Finally, the GCs were immunoreactive to the neurotrophin systems BDNF/TrkB, NGF/TrkA, and NT-3/TrkC, but not always colocalized. The distributions of cellular markers: anti-Chat, anti-Opsin, Parvalbumin, and s100p were perfectly overlapping concerning their specificity. Comparison between the neurotrophin/tyrosine kinase receptor system and specific marker distribution patterns is shown in Figure 7 and Table 1.

## 3. Discussion

According to the latest WHO estimates, 285 million people worldwide suffer visual impairment, and most of the major eye diseases are age-related. As life expectancy rises worldwide, the prevalence of age-related visual impairments has also seen a notable increase. The retina is one of the eye structures that undergoes various structural and functional changes with advancing age. Aging is frequently accompanied by retinal degeneration phenomena commonly associated with conditions such as age-related macular degeneration (AMD) and the progressive degeneration of photoreceptor cells, impaired retinal pigment epithelium function, and alterations in the vascular network [59]. Hence, visual impairment due to retinal degeneration constitutes one of the significant concerns in the aging population, affecting its quality of life and the healthcare system [60,61]. Aging impacts not only the function of the visual system but also its ability to protect and repair damaged and/or degenerating neurons [34,59,62]. Unfortunately, there is no treatment to curb the neurodegenerative disease of the eye, and the lifespan length of most traditional experimental models has impaired the study of the aging process. Among killifish, *Nothobranchius* spp. has a relatively short life cycle compared to other vertebrate models and has several aging features already described for humans [63], which makes it an excellent aging model to fill this gap [30]. In addition, it has been observed that *Nothobranchius’* central nervous system, including the visual system, shows typical aging features [29,64,65,66,67,68]. Therefore, *Nothobranchius* spp. seems to be the ideal model for studying cellular and molecular age-dependent changes to understand and deal with neurodegenerative events effectively. Moreover, the visual system is considered an important tool to investigate the brain overall, both in mammals and fish, since the retina is an integral part of the central nervous system [69]. It has been shown that the pathological processes occurring in the retina indicate similar processes occurring in the central nervous system and vice versa [56,57,58,70]. For instance, alterations in neurotrophin balance are involved in both AMD and retinopathy and in Alzheimer’s disease pathogenesis [71,72,73,74]. The research on the aging of the eyes, particularly of the retina, represents a promising strategy for studying neurodegenerative diseases [75], as it is considered a window to the brain. As a sensory system, retina development [76,77] and maintenance [78] are controlled by neurotrophins, in particular, brain-derived neurotrophic factor (BDNF) [7]. Neurotrophins and their receptors have been shown to be evolutionarily conserved and they have been detected in different vertebrates from fish to mammals, including humans [4,79,80,81,82].

They play a key role in guiding the maintenance, regulation, and neurogenesis of teleosts and the human nervous system, the sensory organs, and, among them, the retina.

Being that the retina is an integral part of the nervous system and given the established link between neurotrophins and the overall well-being of the nervous system, it is plausible to hypothesize a relationship between neurotrophin and retinal neurodegeneration during aging. As aging progresses, the decline in neurotrophin support could contribute to the degenerative changes observed in the retina, such as those associated with age-related macular degeneration and retinal degeneration due to the loss of retinal cells [27,28,29,30,31,32,57,58,59,60,61]. Consequently, scientific research focused on the potential neuroprotective and therapeutic role of neurotrophins and their receptors in treatment of retinal diseases and, neurodegeneration [10]. Indeed, studies have reported that intraocular administration of NTs promotes the survival of GCs and axons after injuries [83,84,85,86,87,88,89,90] and, in particular, the intraocular administration of BDNF has shown a protective action on photoreceptors in retinal degeneration and retinal detachment [91]. Hence, the relevance of this field of investigation is intuitive, and choosing appropriate models that mimic human physiological conditions is crucial for the translational relevance of findings. In this context, *N. guentheri* represents a suitable experimental model because of the functional and morphological similarities of it and the human retina and its emergent role in aging studies. Moreover, the *N. guentheri* retina has demonstrated properties of neurogenesis and regeneration [1].

However, evidence of NTs-Trks system expression in the retina of this experimental model is still scarce. According to the authors’ knowledge, only TrkB has been localized in the retina of one species of the *Nothobranchius* genus, *N. furzeri* [26,91,92,93].

Therefore, this work aimed at studying the localization of the neurotrophins BDNF, NGF (nerve growth factor), NT-3 (neurotrophin-3), and tyrosine protein kinase receptors type A (TrkA), TrkB, and tyrosine protein kinase receptors type C (TrkC) neurotrophin receptors, in the retina of adult *N. guentheri* as a first step in assessing the suitability of this model organism for translational medicine studies.

This study shows, for the first time, that NTs/Trks systems are widely detectable in the adult *N. guentheri* retina, mainly in the RPE, PRL (photoreceptor layer inner and outer segments), OPL (outer plexiform layer), and several populations of ACs (amacrine cells), BCs (bipolar cells), and HCs (horizontal cells) in the INL and in the GCL (ganglion cell layer). The present study’s data agree with the localization of NTs/Trks in the retina of other species, including humans [83]. In the present study, NGF neurotrophin and TrkA receptors were observed in RPE, PRL, and OPL, in several populations of ACs, in BCs, and in several GCs populations. This data overlaps with the location of NGF and TrkA retina of mice [94].

In the retina of *N. guentheri* Trks receptor types A, B, and C were immunolocalized in the external and internal segments of the photoreceptors. In addition, several populations of ACs identified by morpho-topographic approach showed immunoreactivity to neurotrophin receptors type A, B, and C. BCs were always immunopositive to Trks and HCs TrkB and TrkC immunoreactive. Finally, TrkB and TrkC are immunolocalised in different GC populations recognizable by using a morpho-topographic approach and have not shown double staining with neurotrophins. The above-mentioned results partly overlap with the data concerning the mouse retina [95,96,97] and other teleosts, including zebrafish.

In this study, the authors found that the location of Trks receptors in *N. guentheri*’s retina overlaps with the distribution of BDNF in other teleosts [60,82,85,86].

In addition, the results of this study show the presence of TrkA, TrkB, and TrkC receptors in GCs, where BDNF had already been detected by Gatta et al. [71]. The present results support Gatta’s hypothesis [71] on the mode of autocrine action of BDNF on GCs. in addition, BDNF was observed in the *N. guentheri* retina in the RPE, in the external and internal segment of photoreceptors layer, and in ACs in the INL. Localizing the BDNF is intriguing because it has been demonstrated that its decrease is associated with age-dependent macular degeneration and retinopathy [10,71,84,85,86,87,88,89,90,91,92,93,94,98,99,100,101].

Based on our knowledge, no data are known in regard to the localization of neurotrophin NT-3 in the retina of *N. guentheri*. In this study, the authors show, for the first time, that the prolongations of the RPE, the external and internal segments of the photoreceptors, BCs, some ACs, and GCs are immunopositive. These data are similar to what is recognized in other species such as the pigeon (*Columba livia* [96], lizard (*Gallotia galloti*) [97], frog [102], chick [17,103], and mice [104]. In addition, Das and colleagues [105] investigated BDNF/TrkB and NT-3/TrkC systems and observed localization in the external segment of the PRL, OPL, INL, IPL, and GCL, as shown in this work in the *N. guentheri* retina.

Finally, in the retina di *N. guentheri* BDNF and NT-3 neurotrophins and neurotrophin receptors Trks (A, B, C) were found immunolocalized in the pigmented epithelium, a retinal layer relevant to the study of age-induced damage. Indeed, research demonstrates that RPE changes are a typical characteristic of the aging retina [49]. The thinning of the retinal layers and a decrease in the pigment epithelium layer typical in old specimens of *Oryzias latipes* and *D. rerio* [94,106] occur in a short time before the end of the *N. guentheri* life cycle. This evidence makes the retina of the annual killifish an excellent model in the biomedical research of age-dependent pathologies [1].

To compare the localization of neurotrophins and neurotrophin receptors A, B, and C in different retinal cell layers of the different models with humans, see Table 2.

The data of the present investigation, together with the known evidence on *N. furzeri* [26,91,93,120,121] and other species [99,102,122] confirm the fundamental role of the neurotrophins/receptors system in the maintenance and modulation of excitatory input in retinal neurons. Consequently, it might also suggest a mode of autocrine action of the NT/Trks system in the retina of the adult *N. guentheri*.

## 4. Materials and Methods

### 4.1. Fish and Tissue Treatment

In this study, paraffin embedded tissue of *Nothobranchius guentheri* from previous studies were used [123]. Adult specimens of *N. guentheri*, (discovered dead of unknown causes) 1-year-old, 1 male, and 2 females, from ornamental aquariums (freshwater, 22 °C, pH 6.8–7.0) were used. The heads were quickly removed, fixed in 4% paraformaldehyde (Sigma-Aldrich, Inc., St. Louis, MO, USA # 158127) in phosphate-buffered saline (PBS, Sigma-Aldrich, Inc., St. Louis, MO, USA # P4417) 0.1 m (pH = 7.4) for 12–18 h, dehydrated through graded ethanol series, clarified in xylene, for paraffin wax (Bio-Optica Milano S.p.a Milano, Italy # 08-7910) embedding.

### 4.2. Optical Microscopy

Included tissues of *N. guentheri* were cut into 7 μm thick serial sections and collected on gelatin-coated microscope slides [123,124].

Then, serial sections were deparaffinized and rehydrated, washed in distilled water, and stained with Hematoxylin-Eosin (Hematoxylin nuclear staining Bio-Optica Milano S.p.a Italy cat. # 05-M06012. Eosin Y cytoplasmic staining Bio-Optica Milano S.p.a Italy cat. # 05-M10002). At the end, stained sections were examined under a Leica DMRB light microscope equipped with Leica MC 120 HD camera (Leica Application Suite LAS V4.7).

### 4.3. Immunohistochemistry

To analyze the localization of neurotrophins (NTs) and tyrosine protein kinase receptors (Trks) in *N. guentheri* retina, some serial slides were deparaffinized and rehydrated, finally washed in PBS. The sections were incubated in 0.1% Triton X100 (Sigma-Aldrich, Inc., St. Louis, MO, USA cat. #X100) PBS solution to permeate the membranes, after incubated in a 0.3% hydrogen peroxide solution (H_2_O_2_ Sigma-Aldrich, Inc., St. Louis, MO, USA cat. # 1085971000) to prevent the activity of endogenous peroxidase. The 25% fetal bovine serum solution (Sigma-Aldrich, Inc., St. Louis, MO, USA cat. #F7524) was then added to the rinsed sections. Sections were incubated overnight at 4 °C in a humid chamber with primary antibodies (steps below). Representative sections were incubated with appropriately preabsorbed antisera as mentioned above to provide negative controls. In these circumstances, there was no evidence of positive immunostaining.

#### 4.3.1. TrkA, TrkB, TrkC, Anti-Opsin, Anti-Chat, Parvalbumin and s100p Immunofluorescences

To identify anti-neurotrophin receptors in retinal cells of *N. guentheri* some serial sections were incubated with tyrosine protein kinase receptors type A (TrkA), tyrosine protein kinase receptors type B (TrkB), tyrosine protein kinase receptors type C (TrkC) (for details see Table 1). Moreover, some representative sections have been incubated with anti-Opsin, anti-Chat, Parvalbumin, and S100p antibodies, recognized as specific markers for retinal cells (for details see Table 1). After rinsing in PBS solution, the sections were incubated for 1 h with a fluorescent secondary antibody Anti-mouse IgG (H+L) Alexa Fluor 488 and Anti-rabbit IgG (H + L) Alexa Fluor 488 (for details see Table 1) at room temperature in a dark humid chamber. Washing, and mounting using Fluoromount Aqueous Mounting Medium (Sigma-Aldrich, Inc., St. Louis, MO, USA cat. #F4680 manufacturer’s notice) were the final steps.

#### 4.3.2. NTs/Trks System Double Immunofluorescences

To investigate the immunolocalization of the NTs/Trks system in n guentheri retina polyclonal BDNF brain-derived neurotrophic factor and NGF (nerve growth factor) were used in double-labelled experiment with monoclonal TrkB and TrkA, respectively, and monoclonal NT-3 (neurotrophin-3) was used in double-labelled experiment with monoclonal TrkC (for details see Table 1). After rinsing in PBS solution, the sections were incubated for 1 h with a fluorescent secondary antibody anti-rabbit alexa fluor 594 and anti-mouse alexa fluor 488 (for details see Table 3) at room temperature in a dark humid chamber. Washing, and mounting using Fluoromount Aqueous Mounting Medium (Sigma-Aldrich, Inc., St. Louis, MO, USA cat. #F4680 manufacturer’s notice) were the final steps.

#### 4.3.3. Confocal Laser Scanning Microscope

A Zeiss LSMDUO confocal laser scanning microscope with META module (Carl Zeiss MicroImaging GmbH, München, Germany) was used to detect the immunofluorescence, and Zen 2011 (LSM 700 Zeiss software ZEN 3.7) was employed to process the images [125,126,127]. Each image was rapidly acquired to minimize photodegradation.

### 4.4. Statistical Analysis

ImageJ software was used to evaluate microscope fields collected randomly. One-way ANOVA was used to examine the statistical significance of the quantity of retinal pigment epithelium (RPE), PRL (photoreceptor layer inner and outer segments), OPL (outer plexiform layer), Acs (amacrine cells), IPL (inner plexiform layer), BCs (bipolar cells), HCs (horizontal cells) and GCs (ganglion cells) detected by BDNF, NGF, NT-3, TrkA, TrkB, TrkC, anti-Opsin, anti-Chat, Parvalbumin and, s100p. SigmaPlot version 14.0 (Systat Software, San Jose, CA, USA) was used to conduct the statistical analysis. An unpaired Z test was also performed. The information was given as mean values with standard deviations (Δσ). Values of *p* below 0.05 were considered statistically significant in the following order *** *p* < 0.001, ** *p* < 0.01, * *p* < 0.05.

## 5. Conclusions

In conclusion, our study provides new insights into the localization of neurotrophins and their specific receptors in the retina of *Nothobranchius guentheri*, showcasing its potential as an experimental model for investigating retinal aging. Furthermore, the conservation of neurotrophin signaling pathways in *N. guentheri* suggests its relevance as a translational model for studying retinal aging in humans. However, while our findings offer valuable insights, they just represent a starting point in comprehensively characterizing retinal aging in *N. guentheri*, future studies are needed to better understand the expression patterns of the NTs/Trks system during development, the aging process, and/or in transgenic *N. guentheri* models for neurodegenerative diseases. It is still necessary to elucidate the functional consequences of NT-Trk system alteration in the aged retinas of *N. guentheri* and explore potential therapeutic interventions to mitigate age-related retinal degeneration.

## Figures and Tables

**Table 1:** Mean data ± standard deviation (∆σ) of immunopositivity of: RPE, PRL (inner and outer segments), OPL, ACs, BCs, HCs, IPL, and GCs detected by BDNF, NGF, NT-3, TrkA, TrkB, TrkC, and compared with anti-Opsin, anti-Chat, Parvalbumin and, s100p. The statistical analysis shows a different distribution pattern of the antibodies used in this study in cellular layer of *N. guentheri* retina. Mean values ± standard deviation (Δσ). (ꟷ) No value. Statistical significance: *** *p* < 0.001, ** *p* < 0.01, * *p* < 0.05.

	Antibodies Investigated						Specific Antibodies for Retinal Cells			
	BDNF	TrkB	NGF	TrkA	NT-3	TrkC	Opsin	Chat	Parv	S100p
Mean ± ∆σ in RPE	28.4 ± 2.15 ***	27.8 ± 1.95 *	ꟷ	28.6 ± 2.45 ***	25.9 ± 2.46 ***	25.9 ± 2.85 ***	27.2 ± 1.24 **	26.3 ± 1. 83 ***	26.5 ± 2.57 ***	28.8 ± 5.11 ***
Mean ± ∆σ in PRL (outer segment)	27.7 ± 2.49 *	ꟷ	27.7 ± 2.49 *	ꟷ	ꟷ	25.3 ± 3.13 ***	26.9 ± 3. 82 *	26.8 ± 2. 40 **	25.8 ± 2.35 ***	26 ± 3.66 ***
Mean ± ∆σ in PRL (inner segment)	26.4 ± 2.9 **	26.3 ± 2.86 **	27.9 ± 2.80 *	28.4 ± 2.74 ***	25.2 ± 3.6 **	ꟷ	27.1 ± 4. 19 **	ꟷ	23.7 ± 3.95 ***	27.8 ± 5.97 ***
Mean ± ∆σ in OPL	26 ± 3.66 ***	25.3 ± 3.13 ***	25.8 ± 2.35 ***	26.5 ± 2.57 ***	26.2 ± 2.08 ***	25.4 ± 2.15 **	ꟷ	ꟷ	ꟷ	ꟷ
Mean ± ∆σ of ACs	27.8 ± 2.31 *	26.9 ± 2.58 ***	27.7 ± 2.49 *	27.9 ± 2.79 *	ꟷ	26.8 ± 2. 52 ***	ꟷ	27.9 ± 3. 04 *	26.5 ± 5.48 ***	26.7 ± 4.64 ***
Mean ± ∆σ of BCs	ꟷ	26.9 ± 2.36 ***	26.5 ± 2.57 ***	27.8 ± 3.65 *	26.5 ± 2.57 ***	27.7 ± 2.14 ***	ꟷ	ꟷ	27.7 ± 5.36 ***	25.8 ± 4.6 ***
Mean ± ∆σ of HCs	ꟷ	27.7 ± 2.49 *	ꟷ	ꟷ	ꟷ	25.8 ± 2.67 ***	ꟷ	ꟷ	ꟷ	26.3 ± 4 **
Mean ± ∆σ in IPL	ꟷ	ꟷ	ꟷ	ꟷ	ꟷ	ꟷ	ꟷ	ꟷ	27.4 ± 4.8 **	26.5 ± 5.12 ***
Mean ± ∆σ in GCs	27.2 ± 1.24 **	26.9 ± 1. 42 **	26 ± 2.89 *	26.7 ± 3. 40 ***	27.9 ± 2.38 *	27.2 ± 2.62 **	ꟷ	ꟷ	26.7 ± 4.42 ***	27.4 ± 4.84 ***

**Table 2:** Comparison of different species’ neurotrophin (BDNF, NGF, and NT-3), and tyrosine kinase receptor (TrkA, TrkB, TrkC) localization and expression in the retina layers of *N. guentheri*.

Species		N. guentheri *						Zebrafish						Mouse						Human					
Antibodies		BDNF	NGF	NT-3	Trks			BDNF	NGF	NT-3	Trks			BDNF	NGF	NT-3	Trks			BDNF	NGF	NT-3	Trks		
					A	B	C				A	B	C				A	B	C				A	B	C
Cell layers	RPE	+	n/a	+	+	+	+	+	+	−	+	+	+	+	+	n/a	+	+	−	+	n/a	n/a	+	+	−
	R.							[10,81,105,107,108,109,110,111,112]						[17,96,101]						[97,113]					
	PRL	+	+	+	+	+	+	+	+	−	+	+	+	+	+	+	+	+	+	+	n/a	n/a	+	+	+
	R.							[10,81,110,111,112]						[95,101,108,114]						[97,115]					
	OPL	−	−	−	−	−	−	+	+	−	+	+	+	+	n/a	+	+	−	+	+	n/a	n/a	+	+	+
	R.							[10,81,110,111,112]						[95,108]						[97,115]					
	INL	+	+	+	−	−	−	+	+	−	+	+	+	+	+	+	+	+	−	+	n/a	n/a	+	+	+
	R.							[10,81,110,111,112]						[95,108,116]						[97,115]					
	HCs	−	−	−	−	−	−	+	+	−	+	+	+	+	+	n/a	+	+	+	n/a	n/a	n/a	+	+	+
	R.							[10,81,110,111,112]						[95,108]						[97]					
	BCs	−	+	+	+	+	−	+	+	−	+	+	+	+	+	n/a	−	−	−	n/a	n/a	n/a	n/a	n/a	n/a
	R.							[10,81,110,111,112]						[95,101,108,114,116]											
	ACs	+	+	+	+	+	+	+	+	−	+	+	+	+	n/a	+	+	−	−	+	n/a	n/a	+	+	+
	R.							[10,110,111,112,117]						[95,108]						[97,118]					
	IPL	−	−	−	−	−	−	+	+	−	+	+	+	+	n/a	n/a	+	+	+	+	n/a	n/a	+	+	+
	R.							[10,81,102,110,111,112]						[95,108]						[97,119]					
	GCL	+	+	+	+	+	+	+	+	−	+	+	+	+	+	+	+	+	+	+	+	+	+	+	+
	R.							[10,81,102,110,111,112]						[17,95,96,107,120]						[97,121,122,123]					

(*) these data refer to the sample of the present study. (+) positive for the considered antibody; (−) negative for the considered antibody; (n/a) references data not known, to the best of the authors’ knowledge. References (R.), Brain-derived neurotrophic factor (BDNF), nerve growth factor (NGF), neurotrophin-3 (NT-3), tyrosine protein kinase receptors type A (TrkA), tyrosine protein kinase receptors type B (TrkB), tyrosine protein kinase receptors type C (TrkC), retinal pigment epithelium (RPE), photoreceptor layer (PRL), outer nuclear layer (ONL), outer plexiform layer (OPL), inner nuclear layer (INL), amacrine cells (ACs), bipolar cells (BCs), horizontal cells (HCs), inner plexiform layer (IPL), ganglion cell layer (GCL).

**Table 3:** Details of antibodies.

Primary Antibodies	Supplier	Catalogue Number	Source	Dilution	Antibody ID
BDNF	Sigma-Aldrich, Inc., St. Louis, MO, USA	AB1534SP	rabbit	1:100	AB_90748
NGF	Sigma-Aldrich, Inc., St. Louis, MO, USA	AB1526	rabbit	1:100	AB_90733
NT-3 (A4)	Santa Cruz Biotechnology, Inc., Dallas, TX, USA	sc-518099	mouse	1:100	
TrkA (Y32Ex)	Santa Cruz Biotechnology, Inc., Dallas, TX, USA	sc-80398	mouse	1:100	AB_1130726
TrkB (F-1)	Santa Cruz Biotechnology, Inc., Dallas, TX, USA	sc-377218	mouse	1:100	AB_2801499
TrkC (798)	Santa Cruz Biotechnology, Inc., Dallas, TX, USA	sc-117	rabbit	1:100	AB_632560
Anti-Opsin Clone RET-P1	Sigma-Aldrich, Inc., St. Louis, MO, USA	O4886	mouse	1:100	AB_260838
Anti-Chat	Sigma-Aldrich, Inc., St. Louis, MO, USA	AMAB91130	mouse	1:100	AB_2665812
Parvalbumin clone PA235	Sigma-Aldrich, Inc., St. Louis, MO, USA	P-3171	mouse	1:1000	AB_2313693
S100p	Dako Agilent, Santa Clara, CA, USA	Z0311	rabbit	1:100	AB_10013383
**Secondary Antibody**	**Supplier**	**Catalogue Number**	**Source**	**Dilution**	**Antibody ID**
Anti-rabbit IgG (H + L) Alexa Fluor 594	Molecular Probes, Invitrogen, Waltham, MA, USA	A32754	Donkey	1:300	AB_2762827
Anti-rabbit IgG (H + L) Alexa Fluor 488	Molecular Probes, Invitrogen, Waltham, MA, USA	A-11008	goat	1:300	AB_143165
Anti-mouse IgG (H + L) Alexa Fluor 488	Molecular Probes, Invitrogen, Waltham, MA, USA	A-11001	goat	1:300	AB_2534069

## Data Availability

All data presented this study are available from the corresponding author, upon responsible request.

## Abbreviations

neurotrophins (NTs), tyrosine protein kinase receptors (Trks), brain-derived neurotrophic factor (BDNF), nerve growth factor (NGF), neurotrophin-3 (NT-3), tyrosine protein kinase receptors type A (TrkA), tyrosine protein kinase receptors type B (TrkB), tyrosine protein kinase receptors type C (TrkC), retinal pigment epithelium (RPE), photoreceptor layer (PRL), outer nuclear layer (ONL), outer plexiform layer (OPL), inner nuclear layer (INL),amacrine cells (ACs), bipolar cells (BCs), horizontal cells (HCs), inner plexiform layer (IPL), ganglion cell layer (GCL), ganglion cells (GCs).
